# Generating a novel and reliable human iPSC‐derived midbrain organoid model of sporadic progressive supranuclear palsy

**DOI:** 10.1002/alz.093263

**Published:** 2025-01-03

**Authors:** Grace A. Selecky, Kristen R. Whitney, Margaret M. Krassner, Alessandra Cervera, Claudia De Sanctis, Victoria Flores Almazan, Sean Thomas Delica, SoongHo Kim, Kurt W. Farrell, Thomas D. Christie, Megan A Iida, Lily Sarrafha, Gustavo Parfitt, Ruth H. Walker, Melissa J Nirenberg, Giulietta M. Riboldi, Steven J. Frucht, Tim Ahfeldt, Sally Temple, John F. Crary

**Affiliations:** ^1^ Icahn School of Medicine at Mount Sinai, New York, CA USA; ^2^ Ronald M. Loeb Center for Alzheimer’s Disease, New York, NY USA; ^3^ Icahn School of Medicine at Mount Sinai, New York, NY USA; ^4^ Friedman Brain Institute, New York, NY USA; ^5^ Department of Artificial Intelligence & Human Health, Nash Family Department of Neuroscience, Ronald M. Loeb Center for Alzheimer’s Disease, Friedman Brain Institute, Neuropathology Brain Bank & Research CoRE, Icahn School of Medicine at Mount Sinai, New York, NY USA; ^6^ Department of Pathology, Icahn School of Medicine at Mount Sinai, New York, NY USA; ^7^ James J. Peters Veterans Affairs Medical Center, Bronx, NY USA; ^8^ The Marlene and Paolo Fresco Institute for Parkinson’s and Movement Disorders, NYU Langone Health, New York, NY USA; ^9^ Neural Stem Cell Institute, Rensselaer, NY USA

## Abstract

**Background:**

The accumulation of abnormal tau protein in neurons and glia in the human brain is the defining feature of neurodegenerative diseases known as tauopathies. Progressive supranuclear palsy (PSP), the most common primary tauopathy, is typified by selective vulnerability of dopaminergic neurons and glia in the midbrain leading to an atypical parkinsonian movement disorder. To investigate candidate disease mechanisms underlying PSP, there is a critical need for model systems that more accurately recapitulate the cellular and molecular environment in the human brain. Human induced pluripotent stem cell (hiPSC)‐derived organoid models have emerged as a powerful tool to address this gap.

**Method:**

Skin biopsies were collected from living clinically diagnosed PSP patients or during autopsy. Fibroblasts were cultured and reprogrammed into hiPSCs using Sendai virus. HiPSCs were maintained with StemCultures FGF2 Discs to improve pluripotency and FACS was performed to confirm pluripotency marker expression. To generate midbrain organoids, hiPSCs were seeded into suspension spinner flasks, patterned using pharmacological directed differentiation, and grown for four months. Reliable patterning was confirmed with qRT‐PCR, immunohistochemistry and immunoblot using a panel of cell‐type specific markers. Astrocytes were extracted from mature organoids, cultured, and screened for astrocyte‐specific markers.

**Result:**

Fibroblasts have been banked from twenty‐two PSP patients and seven reprogrammed into hiPSCs. Sporadic case status was determined by Sanger sequencing confirming the absence of a *MAPT* mutation. We found that hiPSCs grown with controlled‐release FGF2 discs were 90‐100% positive for pluripotency markers and negative for off‐target genes. During patterning, organoids displayed morphological and cytoarchitectural patterns consistent with developing neuroectoderm and midbrain. Midbrain neural progenitor and dopaminergic markers such as *FOXA2*, *LMX1A*, and *TH* were positive in a time‐dependent manner, with mature dopaminergic neurons expressing *NURR1* and *GIRK2* detected by day 30. GFAP‐positive astrocytes appeared around day 100. Astrocytes extracted from mature organoids were positive for multiple astrocyte markers.

**Conclusion:**

Sporadic PSP patient hiPSCs reliably differentiate into midbrain dopaminergic organoids and astrocytes, resulting in a sporadic tauopathy model containing key cell types affected in PSP. This cell collection is a valuable resource to investigate candidate mechanisms underlying tauopathy and could provide insight into cell‐type specific disease drivers.

